# Increasing sensitivity of clinical proverb tests for diagnosis of dementia

**DOI:** 10.1002/alz.13688

**Published:** 2024-01-22

**Authors:** Vanja Kljajevic

**Affiliations:** ^1^ Department of Special Needs Education University of Oslo Oslo Norway

Cases of dementia worldwide are estimated to double, if not triple, by 2050.^1^ Since there is no cure for dementia, prevention, early diagnosis and interventions that slow down cognitive decline are critical. However, early diagnosis of dementia remains a challenge. Chakrabarty et al.^2^ propose that certain cognitive tests, such as those targeting pragmatic skills, may facilitate early detection of cognitive decline in dementia. The authors argue that disruption to pragmatic processing may precede a deficit in episodic memory, which is considered a hallmark of unfolding Alzheimer's disease (AD),^3^ and that the hippocampus, which is a locus of early neurodegenerative changes in AD,^4^ also supports pragmatic processing.

The idea that comprehension of figurative language may provide a window into cognitive decline is not new. Since the early 20th century, psychologists, psychiatrists, and neuropsychologists have used proverbs for evaluative and diagnostic purposes.^5^ Proverbs are fixed expressions with figurative meanings that communicate well‐known truths and social and moral norms.^6^ Importantly, different types of proverbs have different internal features,^7^ which allow tapping different cognitive abilities (e.g., analogical reasoning, categorization, metaphoric thought, and other symbolic cognitive processes). Thus, comprehension of proverbs based on metaphorical thought (e.g., *Old love does not rust*), synecdochic (e.g., *Strike while the iron is hot*), metonymic (e.g., *The pen is mightier than the sword*), hyperbolic (e.g., *All is fair in love and war*), and paradoxical proverbs (e.g., *Only the lost is owned forever*) may present different degrees of difficulty to different patient populations, because their figurative meaning may rely on different cognitive processes. Regardless, the internal features of proverbs are largely underutilized in current clinical proverb tests, which typically use a small number of apparently randomly selected items. For example, the Delis–Kaplan Executive Function System (D‐KEFS)^8^ proverb subtest contains eight such items and there are assessment tools that involve only one proverb. This opens the question of whether a test consisting of a balanced number of semantic classes of proverbs would be a more sensitive screening tool for detection of early cognitive decline in neurodegenerative diseases, such as AD.

What sparks interest in Chakrabarty et al.’s (2023) paper is the argument that tests of figurative language might help, as a complementary tool, in early diagnosis of AD. Given the importance of early diagnosis, generating new knowledge on how to develop more sensitive cognitive tests using proverbs and other expressions with figurative meaning is gaining in significance. To develop more sensitive tests of figurative language that could be useful in disentangling typical cognitive aging from preclinical AD, one would need to go beyond frequency, abstractness, and syntactic complexity of these expressions and consider their internal features, as illustrated above by the example from semantic classes of proverbs. Perhaps such tests would challenge recent findings that cognitively healthy elderly people comprehend proverbs as well as young people in certain circumstances,^9^ or even better, despite significant atrophy in the relevant brain areas.^10^ Whether tests of figurative language will in the future have more clinical applicability in early diagnosis and monitoring of cognitive decline in dementia depends critically on improving their sensitivity, which in the case of proverb tests means taking into consideration their internal features, such as semantic class.

## CONFLICT OF INTEREST STATEMENT

The author declares no conflicts of interest. Author disclosures are available in the supporting information.

## Supporting information

Supporting Information
